# Circadian rhythm and daytime variation do not affect intraoperative bacterial sternal contamination and postoperative wound infections following cardiac surgery

**DOI:** 10.1038/s41598-024-78435-x

**Published:** 2024-11-04

**Authors:** Moritz Benjamin Immohr, Yukiharu Sugimura, Michelle Hartmann, Ajay Moza, Payam Akhyari, Ali Aljalloud

**Affiliations:** 1 Department of Thoracic and Cardiovascular Surgery, West German Heart and Vascular Center, Medical Faculty and University Hospital Essen, University of Duisburg-Essen, Hufelandstr. 55, 405147 Essen, Germany; 2 Department of Cardiac Surgery, Medical Faculty, RWTH Aachen University, Aachen, Germany; 3 Department of Cardiology, Nephrology and Internal Intensive Care, Rhein-Maas Hospital, Würselen, Germany

**Keywords:** Cardiac surgery, Wound healing, Deep sternal infection, Circadian rhythm, Daytime variation, Risk factors, Interventional cardiology

## Abstract

Studies have documented various effects of circadian rhythm and daytime variations on the cardiovascular and immune system as well as wound healing. From June to December 2016, *n* = 367 cardiac surgery patients were enrolled. Microbiological swabs from the mediastinum and subcutaneous wound were taken before sternal closure. Patients were assigned to groups based on operation start: morning (*n* = 219) or afternoon (*n* = 135). Bacterial contamination and wound infections were studied in relation to circadian rhythm and daytime variation. We did not observe any difference in mortality (morning: 3.7%, afternoon: 3.0%, *p* > 0.99) and major adverse events (morning: 8.2%, afternoon: 5.9%, *p* = 0.53). In 27.7% of the morning group, at least one positive intraoperative swab was observed, similar to the afternoon group (25.6%, *p* = 0.71). The incidence of positive presternal swabs was 15.6% in the morning compared to 9.1% in the afternoon (*p* = 0.18). About 90% of the germs detected were part of the natural skin flora (e.g., Cutibacterium acnes and Staphylococcus epidermidis). The incidence of sternal wound infections was 7.3% (morning) and 3.0% (afternoon) (*p* = 0.18). We did not find differences in the incidence of intraoperative bacterial sternal contamination, nor postoperative infections, between patients who underwent cardiac surgery in the morning or afternoon.

## Introduction

The cardiovascular system is influenced by circadian rhythm and daytime variation that affect neurohormonal changes, hemodynamic parameters, and myocardial metabolism^1–5^. Several recent studies have focused on the impact of circadian rhythm and daytime variation in cardiology and cardiac surgery^6–11^. However, these studies have reported contradictory results and/or clinically irrelevant effects^6–11^. Nevertheless, it is well known that the human immune system is dependent on a distinct circadian rhythm, including immune cell function, cytokine concentrations, and the ability to deal with bacterial and viral infections^12,13^. In addition, several chronic inflammatory diseases, and even inflammatory responses in the development of ventilator-induced lung injury are based on circadian changes^14,15^. Even the gut microbiome, which modulates several immunological functions and is associated with several pathologies including metabolic syndrome, cardiovascular diseases, and the development of cancer, underlies a distinct intrinsic and extrinsic circadian rhythm which goes far beyond just the intervals of food intake^16,17^. Furthermore, processes in skin repair and wound healing are also subject to the circadian rhythm^16–18^. These effects are related to circadian changes in cytokines, hormones, gene expressions, and microRNAs that regulate macrophages, fibroblasts, keratinocytes, and collagen biosynthesis^18–20^. Deep sternal wound infections are frequently observed after cardiac surgery with median sternotomy and carry a high burden of morbidity and mortality^19,20^. Although several risk factors have already been identified, the impact of circadian rhythm and variation during the day on bacterial sternal contamination and the development of sternal wound infections remains unclear, leaving an important gap in the literature^21–23^.

Circadian rhythm and daytime variants play an important role in cardiovascular medicine, immunology, and wound healing. Therefore, we studied the possible impact on postoperative sternal wound infections after cardiac surgery. We examined intraoperative sternal bacterial wound contaminations and postoperative infections after cardiac surgery with median sternotomy and compared patients with scheduled surgery in the morning and afternoon.

## Results

### Logistic regression analyses of known risk factors for surgical site infections

Table 1 presents the results of univariate logistic binary regression analyses concerning intraoperative bacterial sternal wound contamination and postoperative sternal wound infections. Male sex was identified as a risk factor for positive intraoperative sternal swabs (odds ratio = 2.28, *p* = 0.01), whereas concomitant diabetes mellitus was determined to be a risk factor for postoperative sternal infections (odds ratio = 3.68, *p* = 0.01). Smoking (*p* = 0.09) and undergoing surgery in the morning (*p* = 0.10) were incorporated into additional multivariate analyses for positive swabs and sternal wound infections, respectively (Table 2). However, unlike male sex and diabetes mellitus, these factors were not substantiated as independent risk factors.

**Table 1:** Univariate regression analysis to identify risk factors for positive intraoperative microbiological wound swabs and postoperative sternal wound infections.

Univariate analysis	*n*	Median and (IQR) or frequency (%)	Positive intraoperative swab		Postoperative sternal infection	
			Odds ratio [95% CI]	*p*-value	Odds ratio [95% CI]	*p*-value
Age (years)	354	69.0 (61.0–76.0)	1.00 [0.98; 1.03]	0.91	1.02 [0.97; 1.07]	0.46
Male sex	354	250 (70.6)	2.28 [1.25; 4.17]	0.01	0.97 [0.36; 2.59]	0.95
Body mass index (kg/m^2^)	354	26.9 (24.3–31.0)	0.99 [0.96; 1.03]	0.67	0.99 [0.92; 1.06]	0.79
Diabetes mellitus	354	125 (35.3)	0.68 [0.40; 1.14]	0.14	3.68 [1.43; 9.49]	0.01
Smoking	354	190 (53.7)	0.65 [0.40; 1.06]	0.09	1.06 [0.43; 2.62]	0.90
Cortisone therapy	354	22 (6.2)	1.74 [0.70; 4.35]	0.24	0.78 [0.10; 6.15]	0.82
Duration of surgery (min)	316	270 (210–300)	0.94 [0.77; 1.13]	0.48	1.00 [0.70; 1.43]	0.99
Start of operation at morning	354	219 (61.9)	1.11 [0.68; 1.83]	0.68	2.58 [0.84; 7.89]	0.10
Positive intraoperative wound swab	335	90 (25.4)			0.53 [0.15; 1.87]	0.32

**Table 2:** Multivariate regression analysis to identify independent risk factors for positive intraoperative microbiological wound swabs and postoperative sternal wound infections.

Parameter	Regression coefficient	Standard error	Odds ratio	*p*-value
Positive intraoperative swab				
Male sex	0.81	0.31	2.26	0.01
Smoking	− 0.41	0.25	0.67	0.10
Postoperative sternal infection				
Diabetes mellitus	1.37	0.49	3.92	0.01
Start of operation at morning	1.05	0.58	2.84	0.07

### Baseline characteristics and operative parameters

Table 3 shows the preoperative baseline characteristics and the operative data of the patients. There were no relevant differences in demographic data including age and sex, as well as concomitant diseases. The mean body mass index was greater than 25.0 kg/m^2^ for both groups and approximately one-third of patients suffered from concomitant diabetes mellitus underlying a notable risk of perioperative wound healing disorders. In addition, nicotine abuse was reported for more than 50% of patients in both (*p* = 0.23) groups and preoperative cortisone therapy for 8.2% (morning) respectively 3.0% (afternoon, *p* = 0.07) further increasing the risk for wound healing disorders in the cohort. More than 70% of patients in both groups underwent coronary artery bypass surgery (CABG). However, valvular procedures (isolated or combined with CABG) were performed more frequently in the morning group. While combined procedures were performed more often in the morning (23.3% respectively 14.9% in the afternoon group, *p* = 0.06), emergency surgery was performed more often in the afternoon (17.9% compared to 5.0% in the morning group, *p* > 0.01). Although extracorporeal circulation and total length of operation did not differ between the two groups, we observed an increase in aortic cross-clamp time in the morning group (77.5±33.5 min respectively 70.3±30.8 min in the afternoon group, *p* = 0.03).

**Table 3:** Preoperative characteristics of patients undergoing cardiac surgery with median sternotomy in regard to the daytime variation of the start of the operative procedure. Patients operated in the morning (*n* = 219) were compared to patients who underwent operation in the afternoon (*n* = 135).

Variables	Morning	Afternoon	*p*-value
	(*n* = 219)	(*n* = 135)	
Age, y (SD)	68.3 (10.0)	67.1 (11.3)	0.41
Female sex, n (%)	65 (29.7)	39 (28.9)	0.91
Height, cm (SD)	172 (11)	171 (10)	0.20
Weight, kg (SD)	82.1 (17.9)	82.0 (15.5)	0.78
Body mass index, kg/m² (SD)	28.5 (11.9)	27.9 (4.2)	0.32
Concomitant diseases			
Diabetes mellitus, n (%)	73 (33.3)	52 (38.5)	0.36
Heart failure			0.17
HFpEF, n (%)	164 (77.7)	103 (78.6)	
HFmrEF, n (%)	25 (11.8)	21 (16.0)	
HFrEF, n (%)	22 (10.4)	7 (5.3)	
Atrial fibrillation, n (%)	38 (17.4)	23 (17.0)	> 0.99
Smoking, n (%)	112 (51.1)	78 (57.8)	0.23
Cortisone therapy, n (%)	18 (8.2)	4 (3.0)	0.07
Operative parameters			
Procedure			0.03
CABG, n (%)	125 (57.1)	99 (73.3)	
AVR, n (%)	34 (15.5)	16 (11.9)	
MVR, n (%)	8 (3.7)	5 (3.7)	
CABG + Valve, n (%)	34 (15.5)	9 (3.7)	
Multiple valves, n (%)	6 (2.7)	1 (0.7)	
Other, n (%)	12 (5.5)	5 (3.7)	
Multiple procedures, n (%)	51 (23.3)	20 (14.8)	0.06
Reoperation, n (%)	5 (2.3)	1 (0.7)	0.41
Emergency operation, n (%)	11 (5.0)	24 (17.9)	< 0.01
On-pump surgery, n (%)	205 (93.6)	124 (91.9)	0.53
Extracorporeal circulation, min (SD)	121 (50.5)	115 (54.3)	0.17
Cross-clamp, min (SD)	77.5 (33.5)	70.3 (30.8)	0.03
Duration of surgery, min (SD)	275 (84.0)	268 (80.3)	0.72

*AVR* aortic valve repair/replacement, *CABG* coronary artery bypass grafting, *HFmrEF* heart failure with mildly reduced ejection fraction, *HFpEF* heart failure with preserved ejection fraction, *HFrEF* heart failure with reduced ejection fraction, *MVR* mitral valve repair/replacement, *SD* standard deviation.

### Impact of daytime variation and circadian rhythm on the perioperative outcome

Table 4 shows the impact of daytime variation and circadian rhythm on the perioperative morbidity and mortality. There was no difference in the postoperative mortality (morning: 3.7%, afternoon: 3.0%, *p* > 0.99) and the incidence of major adverse cardiac and cerebrovascular events (MACCE) defined as in-hospital death, postoperative myocardial infarction, cardiac reintervention and stroke (morning: 8.2%, afternoon: 5.9%, *p* = 0.53). However, there were more bleeding complications in the morning than in the afternoon (10.5% respectively 3.7%, *p* = 0.03).

**Table 4:** Postoperative characteristics of patients undergoing cardiac surgery with median sternotomy in regard to the daytime variation of the start of the operative procedure. Patients operated in the morning (*n* = 219) were compared to patients who underwent operation in the afternoon (*n* = 135).

Variables	Morning	Afternoon	*p*-value
	(*n* = 219)	(*n* = 135)	
Postoperative hospital stay, d (SD)	17.1 (15.0)	14.6 (12.8)	0.06
In-hospital mortality, n (%)	8 (3.7)	4 (3.0)	> 0.99
Postoperative morbidity			
MACCE, n (%)	18 (8.2)	8 (5.9)	0.53
Bleeding, n (%)	23 (10.5)	5 (3.7)	0.03
Re-exploration, n (%)	21 (9.6)	7 (5.2)	0.16
Tracheotomy, n (%)	17 (7.8)	5 (3.7)	0.17
New onset atrial fibrillation, n (%)	49 (22.4)	30 (22.2)	> 0.99
Amiodarone therapy, n (%)	43 (19.6)	28 (20.7)	0.89
Electric cardioversion, n (%)	11 (5.0)	6 (4.4)	> 0.99

*MACCE* major adverse cardiac and cerebrovascular events, *SD* standard deviation.

### Bacterial sternal contamination and wound healing disorders in the context of daytime variation and circadian rhythm

Table 5 shows detailed information on intraoperative microbiological wound swabs, possible sternal wound healing disorders, and postoperative laboratory values focusing on infections. In about one of four patients, any kind of positive microbiological result was found in the intraoperative swabs (morning: 27.7%, afternoon: 25.6%, *p* = 0.71). Although isolated positive mediastinal swabs were seldom observed (2.5% for each group), positive subcutaneous swabs (morning: 15.6%, afternoon: 9.1%) as well as positive results in both mediastinal and subcutaneous swabs (morning: 10.6%, afternoon: 11.6%) were observed more frequently. About 90% of the bacteria detected were part of the natural skin flora, with Cutibacterium acnes and Staphylococcus epidermidis being responsible for more than 80% of the positive results in both groups. In fact, there was no difference in the germ spectrum detected in relation to daytime variation of the operation. A total of *n* = 16 patients (7.3%) of the morning and *n* = 4 (3.0%) of the afternoon group developed any type of postoperative sternal wound infections (*p* = 0.18) resulting in an incidence of 5.5% sternal vacuum therapy for the morning group and 2.2% for the afternoon group (*p* = 0.18). However, the vast majority of patients with postoperative sternal wound infections did not have positive intraoperative wound swabs. We did not find patients with intraoperative detection of bacteria not related to the physiological skin flora who developed sternal wound infection during the postoperative course. Furthermore, postoperative laboratory values could also not show any correlation between circadian rhythm and daytime variation and the development of postoperative infectious complications.

**Table 5:** Primary and secondary outcome parameters of patients undergoing cardiac surgery with median sternotomy in regard to the daytime variation of the start of the operative procedure. Patients operated in the morning (*n* = 219) were compared to patients who underwent operation in the afternoon (*n* = 135).

Variables	Morning	Afternoon	*p*-value
	(*n* = 219)	(*n* = 135)	
Any positive intraoperative wound swab, n (%)	57/206 (27.7)	33/129 (25.6)	0.71
Pre-sternal, n (%)	31/199 (15.6)	11/121 (9.1)	
Retro-sternal, n (%)	5/199 (2.5)	3/121 (2.5)	0.41
Pre- and retro-sternal, n (%)	21/199 (10.6)	14/121 (11.6)	
Multiple types of germs	1/57 (1.7)	1/29 (3.3)	> 0.99
Germs of the natural skin flora	54/58 (93.1)	26/30 (86.7)	0.44
Cutibacterium acnes	29/57 (50.9)	15/29 (51.7)	
Staphylococcus epidermidis	24/57 (43.9)	10/29 (34.5)	
Staphylococcus saccharolyticus	2/57 (3.5)	2/29 (6.9)	
Staphylococcus hominis	1/57 (1.8)	0 (0.0)	
Staphylococcus capitisb	0 (0.0)	1/29 (3.5)	0.49
Staphylococcus aureus	1/57 (1.8)	0 (0.0)	
Streptococcus oralis	0 (0.0)	1/29 (3.5)	
Enterococcus faecalis	0 (0.0)	1/29 (3.5)	
Bacillus cereus	1/57 (1.8)	0 (0.0)	
Wound infection			0.18
Sternal, n (%)	16 (7.3)	4 (3.0)	
Intraoperative negative swab	10/16 (62.5)	4/4 (100.0)	
Intraoperative Cutibacterium acnes	2/16 (12.5)	0 (0.0)	0.70
Intraoperative Staphylococcus epidermidis	4/16 (25.0)	0 (0.0)	
Other, n (%)	4 (1.8)	1 (0.7)	
Sternal vacuum therapy, n (%)	12 (5.5)	3 (2.2)	0.18
Laboratory values			
First postoperative day			
Leucocytes, 1/nl (SD)	10.1 (3.5)	10.4 (4.1)	0.60
Platelets, 1/nl (SD)	170 (70)	177 (66)	0.10
C-reactive protein, mg/l (SD)	72.5 (38.1)	51.0 (44.5)	0.12
Procalcitonin, ng/ml (SD)	5.72 (11.31)	3.49 (6.53)	0.63
Discharge			
Leucocytes, 1/nl (SD)	9.5 (3.8)	9.7 (3.3)	0.32
Platelets, 1/nl (SD)	353 (165)	359 (146)	0.36
C-reactive protein, mg/l (SD)	57.3 (40.0)	53.5 (49.0)	0.25
Procalcitonin, ng/ml (SD)	1.02 (7.79)	0.19 (0.29)	0.57
Readmission			
Leucocytes, 1/nl (SD)	9.4 (3.9)	9.4 (3.4)	> 0.99
Platelets, 1/nl (SD)	301 (103)	361 (131)	0.21
C-reactive protein, mg/l (SD)	46.2 (54.2)	66.4 (84.2)	0.90
Procalcitonin, ng/ml (SD)	0.07 (0.04)	0.66 (1.07)	0.21

*SD* standard deviation.

## Discussion

In this paper, we examine the impact of circadian rhythm and daytime variation on the incidence of intraoperative bacterial sternal contamination and postoperative sternal wound infection after cardiac surgery with median sternotomy. As circadian rhythm and daytime variation are correlated to several mechanisms in cardiovascular medicine, immunology, and wound healing, we speculated that it could also have an impact on postoperative sternal wound infections after cardiac surgery. However, contrary to our initial hypothesis, we found no association between intraoperative bacterial sternal contamination/postoperative sternal wound complications and circadian rhythm/daytime variation.

Although we observed slight differences in the procedures performed, their urgency, and postoperative bleeding complications, there were no differences in the relevant outcome parameters. There were no differences with respect to the incidence of postoperative mortality, morbidity, and MACCE. On the contrary, Montaigne et al. reported an improved outcome for patients who underwent a surgical aortic valve replacement in the afternoon compared to the morning^6^. However, in line with our study, several other groups could not prove these results^7–11^. These studies included a variety of different cardiac surgical procedures, as well as patients with propensity score, multicenter data, and even meta-analysis^7–11^. Therefore, it seems reasonable that we did not find differences in postoperative morbidity, mortality, and MACCE as well. In contrast to these previously reported studies, the primary objective of the study was to examine the effects of circadian rhythm and daytime variation on the postoperative development of complications of the postoperative sternal wound. However, we also did not find differences in the incidence of intraoperative bacterial contamination, germs detected, postoperative wound infections, and the need for sternal vacuum therapy.

It has been described that circulating immune cells increase during nighttime and decrease during the day in humans^12,13^. Meanwhile, the concentration of pro-inflammatory cytokines reaches a peak in the morning^12,13^. Furthermore, studies in mice showed increased vulnerability and decreased survival if exposed to pathogens and bacterial endotoxins during the beginning of their active phase, which would correspond to the morning group in our study^12,13^. In line with this, Felten and colleagues also described an association between the development of ventilator-induced lung injury and mechanical ventilation at the beginning of the active phase in mice^14^. However, we did not find differences in the development of postoperative infections nor alteration in postoperative laboratory values including leukocyte counts, c-reactive protein and procalcitonin concentration with respect to circadian rhythm and daytime variation in our study. The effect of circadian rhythm on postoperative wound healing remains controversial, with very limited data available. While Cable et al. described increased wound healing in hamsters if the wound was administered during the active phase of the animals than during the inactive phase, the effect vanished if the circadian rhythm was interrupted^17^. On the contrary, another group found better postoperative healing of colon anastomoses in animals with disruption of the regular rhythmic day and night^18^. In our clinical study, we did not observe differences in the development of postoperative infectious wound healing disorders, but further studies are needed to strengthen our knowledge in this context, especially as the current literature is limited to animal studies^17,18^.

Although we did not find differences with respect to circadian rhythm and daytime variation, we were able to report detailed data on intraoperative bacterial contamination of sternal wounds during cardiac surgery. By taking microbiological wound swabs before sternal closure, we found an incidence of more than 25% of positive swabs in our study cohort. About 90% of the positive samples revealed germs of the natural skin flora. Although these results appear very high at first glance, they are comparable to those of the literature^21–26^. Nandyala and Schwend reported 23% positive swabs with 69% Cutibacterium acnes in children with spinal deformity surgery^21^. For orthopedic surgery, between 15 and 35% of positive samples have been reported with a similar germ spectrum with predominance of the natural skin flora^22–24^. Finally, in the field of cardiac surgery, an incidence of about 9% positive swabs for coagulase-negative staphylococci and 14% for Cutibacterium acnes have been previously reported before the closure of the saphenectomy wound^25^. Furthermore, recently a rate of more than 70% of positive swabs has been described for subcutaneous tissue of sternal wounds^26^. Similarly to the available literature, we found no correlation between the detection of positive intraoperative wound swabs and the development of wound infections in our cohort^21–25,27^. In fact, only 30% of patients with postoperative sternal wound infections had a positive intraoperative wound swab. In orthopedic surgery, Santoshi et al. even reported that only 4.4% of patients with positive intraoperative wound swabs developed surgical site infection and the relative risk of surgical site infections in patients with positive intraoperative swabs was 0.41^27^. These findings suggest that most wound infections develop in the postoperative course and postoperative wound care could have a tremendous impact on the development of sternal wound infections. Surveillance of postoperative wound contaminations by periodic follow-up swabs during the later course could help to assess potential surgical site infections and could easily be implemented in the clinical routine.

The cumulative incidence of sternal wound infections was approximately 6% in our cohort, and approximately 4% required temporary sternal vacuum therapy. This is slightly higher compared to large registry data that report instances of sternal wound infections after cardiac surgery in 2-3% of cases^19,20,28–30^. However, these data only report deep sternal wound infections, while we were unable to differentiate between superficial and deep sternal wound infections in our study due to the retrospective nature of the analyses.

Surgical site infections have a tremendous impact on the postoperative recovery of patients after surgery. Therefore, in addition to identifying risk factors, potential biomarkers seem to be a promising new perspective and should be implemented in the surgical routine. Recently, serum Butyrylcholinesterase has been described as a novel biomarker for postoperative surgical site infection in colorectal surgery^31^. Furthermore, other studies have reported the ratio of c-reactive protein to lymphocyte, neutrophil to lymphocyte ratio, platelet to lymphocyte ratio, and procalcitonin and prealbumin as early predictive markers for the development of surgical site infections after different types of surgery^32–35^. Although none of these studies has yet included cardiac surgery and sternal infections, routinely periodic measurements of biomarkers in addition to microbiological wound swabs in the postoperative course can help prevent emerging wound infections before they become clinically relevant^31–35^.

### Limitations

The original collection of the data presented was carried out a relatively long period ago for a different purpose. Therefore, the study has a retrospective observational design that limits the validity of the results. Additionally, we must mention the single center character with limited available patient data sets. Furthermore, we do not have detailed information on perioperative hormone homeostasis of the patients, which is necessary to investigate specific mechanisms of circadian rhythm and immune system. Especially measurements of the cortisol level of the patients would have strengthened the validity of the study and will provide a starting point for future research. Although the difference in prevalence of preoperative cortisone therapy in the two groups was only numerically and did not reach statistical significance, it cannot be completely ruled out due to the small size of the group and therefore acts as a possible confounder of the study. Finally, we only compared patients that were operated in the morning or afternoon and excluded patients with nighttime surgeries.

The median sternotomy still represents the standard access for most procedures in cardiac surgery. However, it carries a risk of wound healing complications, especially as a high number of patients in cardiac surgery suffer from relevant risk factors for impaired wound healing. Circadian rhythm and daytime variation have been reported to affect not only the cardiovascular system, but also the immune system and wound healing. Therefore, we examine the impact of circadian rhythm and daytime variation on bacterial wound contamination and postoperative infections in patients undergoing cardiac surgery with median sternotomy. Although we found a surprisingly high incidence of intraoperative bacterial wound contaminations, we were unable to prove any correlation with circadian rhythm and daytime variation. In addition, we could also not prove a correlation between intraoperative bacterial wound contamination and postoperative sternal wound infection. Consequently, in times of high standards of intraoperative hygiene, the clinical implications of circadian rhythm and daytime variations on wound infections seem to remain small.

## Methods

### Human ethics and consent to participate

The ethical approval of the study was granted by the local ethics committee (local trial approval and registration number: EK 151/09 version 1.2.3, March 2016). The study followed the principles of the Declaration of Helsinki, and all patients gave their written informed consent for the scientific use of anonymized patient data before inclusion.

### Patients and study design and objectives

**Fig. 1:**
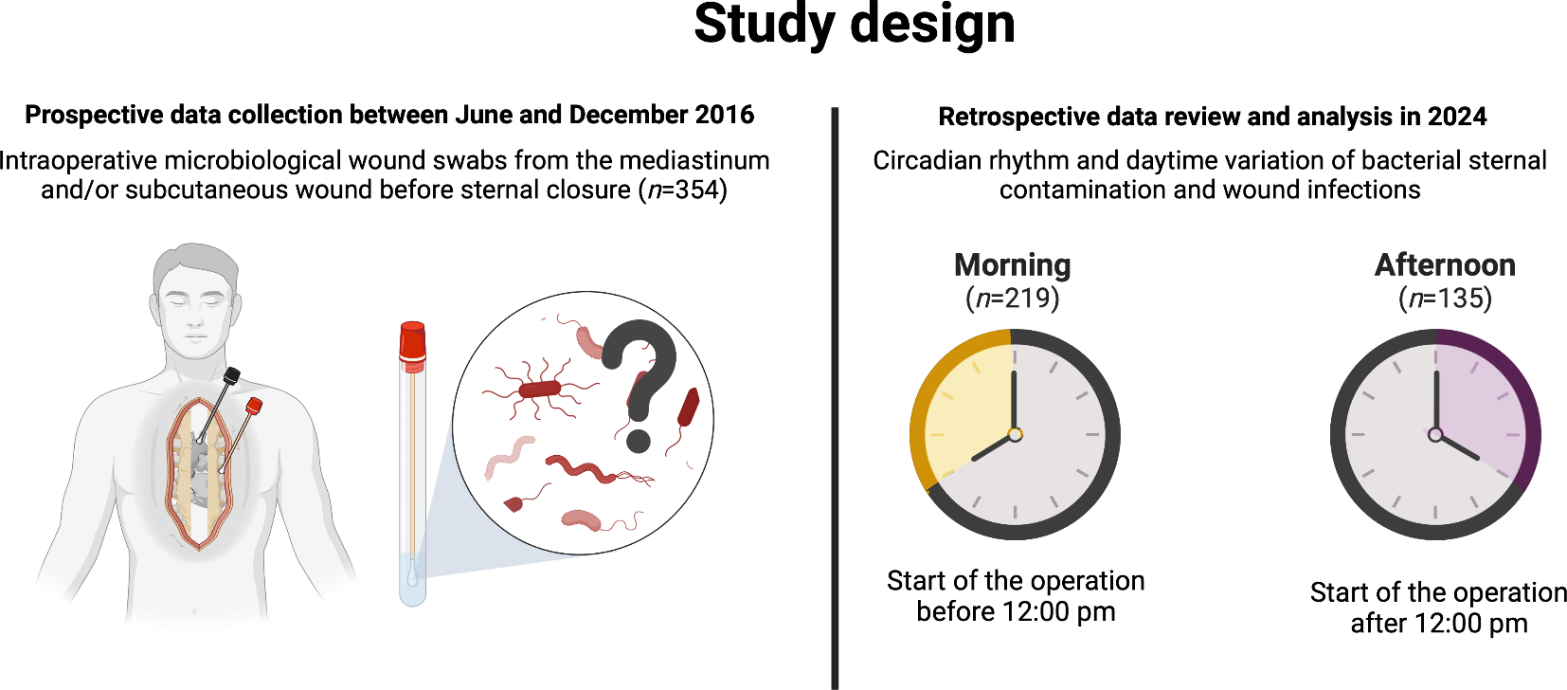
Data collection was carried out in 2016 for a different purpose. Data were reviewed to examine potential effects of circadian rhythm and daytime variation on intraoperative bacterial contamination of sternal wounds retrospectively in 2024. A total of *n* = 354 patients were included and compared in terms of the start time of the surgical procedure (morning (before 12:00 pm), *n* = 219; afternoon (after 12:00 pm), *n* = 135).

Figure 1 gives a brief overview of the study design. Between June and December 2016, all adult patients undergoing cardiac surgery with planned median sternotomy in our department were preoperatively asked permission to participate in a prospective study on complications of sternal wounds. Detailed inclusion and exclusion criteria of the study are listed in Table 6. All patients who gave their informed consent were included. In these patients, microbiological wound swabs were taken from the mediastinum (retrosternal swab), the subcutaneous wound (presternal swab) or both. The swabs were taken directly before sternal closure. All patients were handled following the valid recommendations of the hospital hygiene for cardiac surgery including two times of intraoperative intravenous antibiotic prophylaxis with cephazolin. No additional topic antibiotics (e.g. vancomycin) were routinely applied to the sternal edges. The results of the microbiological probes as well as general patient and procedural data were entered into a study database. In 2024, the database was retrospectively reviewed, and eligible study patients (*n* = 354) were assigned to study groups according to daytime variation of the start of the operative procedure. Patients who started the operation before 12:00 pm (Morning, *n* = 219) were compared with those who underwent cardiac surgery after 12:00 pm (*n* = 135). Patients who underwent surgery at night were not included in the study. Relevant parameters of the patient and outcome were analyzed in a retrospective observational study regime. The incidence of bacterial contamination of the sternum and sternal wound infections was defined as the primary outcome.

**Table 6:** Inclusion and exclusion criteria for patient enrollment of the study.

Inclusion criteria
Cardiac surgery with planned median sternotomy
Age ≥ 18 years
Exclusion criteria
Inability to give informed consent
Surgery at nighttime

### Statistics

Statistical analyses were calculated by SPSS Statistics version 29.0.2.0 (IBM Corporation, Armonk, NY, USA). All results are displayed as mean values with the standard deviation (SD) and percentages of the whole. As group sizes are unbalanced due to the retrospective character of the study, Gaussian distribution was not assumed, and variables therefore compared by either non-parametric two-tailed Mann Whitney U - or Fisher’s exact tests. Furthermore, binary logistic regression analyzes were performed. Parameters with p-value ≤ 0.10 were included in a multivariate binary logistic regression analysis.

## Data Availability

The datasets used and/or analyzed during the current study are available from the corresponding author on reasonable request.
